# Fatal Hepatocellular Carcinoma in a Patient with Occult Hepatitis B Virus Infection following the Administration of R-CHOP for Diffuse Large B-Cell Lymphoma

**DOI:** 10.1155/2012/803298

**Published:** 2012-12-26

**Authors:** Vincenzo Pitini, Massimo Rizzo, Carmela Arrigo, Patrizia Mondello, Giuseppe Altavilla

**Affiliations:** Department of Medical Oncology (Pad. H 5° Piano), University of Messina, Via Consolare Valeria Road 98125 Messina, Italy

## Abstract

Occult hepatitis B (OBI) is caused by a persistent low-level replication of HBV. Like overt HBV infection, OBI can be associated with the integration of HBV sequences into the host genome and has a substantial clinical relevance for patients who are severely immunosuppressed for long durations. We present the case of a patient with a diffuse large B-cell non-Hodgkin lymphoma and OBI who developed a hepatocellular carcinoma with a fulminant clinical course following the administration of rituximab plus CHOP.

## 1. Introduction

Worldwide, chronic hepatitis B virus (HBV) infection is the primary cause of cirrhosis and hepatocellular carcinoma and is considered one of the ten leading causes of death [1]. Traditionally, people with chronic HBV infection have been identified with blood tests for HBV antigens and antibodies. Recently, another group of patients with chronic HBV infection has been identified by sensitive molecular testing for HBV DNA. Members of this group are often referred to as having occult hepatitis B (OBI) which is characterized by undetectable HBsAg, low (<200 IU/mL) or undetectable HBV-DNA levels in serum, with or without anti-HBc positivity, and detectable HBV-DNA in the liver tissue [2]. Etiopathogenically, OBI is caused by persistent low-level replication of HBV resulting from host suppression of HBV replication or from mutant viral strains with defective replication or S-protein synthesis. Like overt HBV infection, OBI can be associated with the integration of HBV sequences into the host genome and recent studies have suggested that OBI has a substantial clinical relevance as an important risk factor accelerating the progression of the liver disease and the development of cirrhosis and hepatocellular carcinoma (HCC) [3, 4]. Furthermore, OBI has a clinical relevance for patients who are severely immunosuppressed for long durations, for example, patients who receive systemic chemotherapy, radiotherapy, or immunotherapy, human immunodeficiency virus-infected individuals, and patients who undergo liver transplantation which may be at risk for the reactivation of HBV infection or fulminant hepatitis failure [5].

We present the case of a patient with a diffuse large B-cell non-Hodgkin lymphoma and OBI (HbsAg, Hbc, and anti-HBs negative) who developed a hepatocellular carcinoma with a fulminant clinical course following the administration of Rituximab plus CHOP.

## 2. Case Report

**Figure 1:**
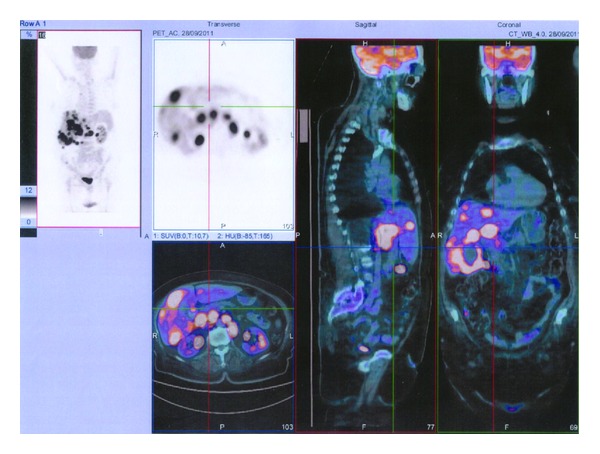
Restaging PET/computed tomography.

**Figure 2:**
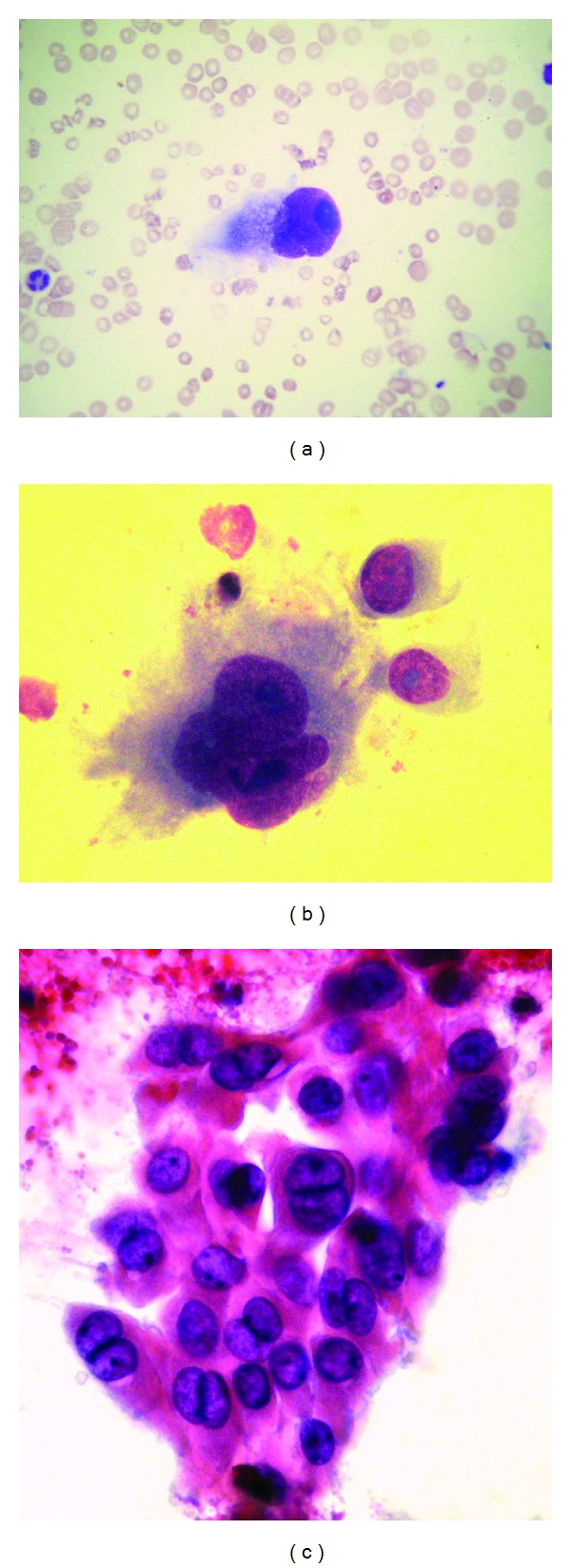
Giant cell with large nuclei and macronucleoli, multinucleated giant cells.

A 58-year-old female had been treated with five courses of rituximab plus CHOP chemotherapy from May 2011 to September 2011 for a diffuse large B-cell lymphoma stage III B. Before the last treatment, the patient presented severe fatigue, abdominal pain, daily temperatures of up to 38.3°C and occasional night sweats. The hematocrit was 28 percent hemoglobin 9.5 g/dL with a white-cell count of 2.32 × 10^9^/L (59% neutrophils and 41% lymphocytes), and platelet counts 51 × 10^9^/L. The levels of bilirubine, alkaline phosphatase, prothrombin time, and creatinine were normal. Albumin was 3.1 g/dL (normal 3.5 to 5 g/dL). Tests for viral hepatitis A, B, and C were negative. The level of ALT was 100 U/L and that of AST was 70 U/L. The level of beta 2-microglobulin was normal, while alpha-fetoprotein was 440 ng/mL. A restaging PET/computed tomography (CT) scan (Figure 1) shows an almost complete resolution of that metabolic activity in lymphnodes involved at diagnosis (right neck, axillae, paratracheal, periaortic, and both inguinal regions) in addition FDG-PET shows several areas of hypermetabolism in the liver (SUV 10.7) indicating the presence of a high grade malignancy. CT scanning revealed multiple hepatic mass (<5 cm.) which shows heterogeneous arterial enhancement after the administration of contrast material. Percutaneous ultrasound guided core biopsies revealed on histologic specimens a poorly differentiated HCC with typical cytological features: giant cells with large nuclei and macronucleoli, multinucleated giant cells (Figures 2(a), 2(b), and 2(c)). A PCR detection of intrahepatic HBV-DNA was performed using described nested PCR methods with primers targeting the S, polymerase, precore-core, and X regions of the HBV genome; unexpectedly, all four HBV regions of HBV DNA were detectable in the liver of this apparent case of cryptogenic hepatocellular carcinoma. Given the size and spread of the tumor with its bilobar involvement, the tumor was deemed not susceptable to surgical resection or ablative therapy and sorafenib therapy was initiated with a dosage of 400 mg orally twice a day. Despite this treatment, however, the patient's condition rapidly worsened and she died after 30 days.

## 3. Discussion 

Chronic hepatitis B (CHB) infection with detectable circulating hepatitis B surface antigen (HBsAg) is a common cause of hepatocellular carcinoma (HCC) in approximately 60% of the world's HCC cases; in addition apparently unidentifiable causes for HCC are frequently HBV related as pointed out in two recent studies which demonstrated that patients with OBI can still develop advanced liver diseases and HCC [3, 6]. Serological findings in patients with OBI vary significantly and, in one review article, approximately 22% of OBI sera were negative, 35% of individuals with OBI were anti-HBs positive, and 42% were anti-HBc positive [4]. The prevalence of OBI in HCC patients varies among different populations, ranging from 16% in the USA, which has a low prevalence of CHB, to 70% in CHB endemic areas like China [7, 8]. HBV contributes to hepatocarcinogenesis through several mechanisms: it can be integrated into the DNA of the host chromosomes, where random insertion adjacent to protooncogenes or tumor-suppressor genes could activate proliferative pathways. In addition, the X HBV protein may itself be oncogenic. Furthermore, active viral replication in the liver causes a necrotizing inflammatory response with the regeneration of hepatocytes that results in an increase in the risk of the accumulation of mutations that contribute to neoplastic transformation. In addition, experimental data shows that HBV positive hepatocytes of transgenic mice exhibit extensive oxidative DNA damage, possibly related to cytokine synthesis which explained their increased sensitivity to malignant transformation by chemical carcinogens, so, there is a possibility that the hepatocarcinogenic risk of OBI increases synergistically during chemotherapy exposition which is able to induce mutagenic and clastogenic DNA damage, including base adducts, replication errors, strand breaks, and cross-links [9]. This is probably relevant in humans exposed to both hepatitis viruses and chemical carcinogens of cancer chemotherapy treatment. The main issue raised by our case is whether OBI may have a clinical relevance in the development and progression of liver oncogenesis in patients exposed to chemotherapeutic treatment and severely immunosuppressed by rituximab for a long duration. Generally speaking, after the introduction of rituximab, the hepatitis B virus reactivation is a well-documented complication that frequently occurs in almost two separate clinical scenarios; the first occurs in patients who have chronic HBV infection in which the diagnosis of the reactivation is based on a detectable serum HBV DNA level in the presence of clinical evidence of hepatitis while, in the second scenario, viral reactivation occurs in patients with a low or undetectable level of HBV replication with or without antibody to B core antigen and anti-HBs. However it is plausible that in both cases long-term immune dysfunction from underlying disease and repeated rituximab treatments are responsible for virus reactivation that may lead to liver failure and death. Why our patient did not develop virus reactivation after treatment while carcinogenesis linked to chemotherapy was highlighted as a late effect associated with exposure to chemotherapeutic drugs remains an unresolved question, even if it seems to be uncommon as recently pointed out in several studies reporting the risk of second malignancy in non-Hodgkin's lymphoma survivors [10–12].

In our opinion, it is difficult to make recommendations regarding the prevention and control of carcinogenesis linked to OBI; perhaps serum microRNA profiles could be a novel biomarker for an early diagnosis of hepatocarcinoma, HBV related [13].
